# Therapeutic Potential of Quercetin in the Management of Type-2 Diabetes Mellitus

**DOI:** 10.3390/life12081146

**Published:** 2022-07-28

**Authors:** Prawej Ansari, Samara T. Choudhury, Veronique Seidel, Akib Bin Rahman, Md. Abdul Aziz, Anika E. Richi, Ayesha Rahman, Umme H. Jafrin, J. M. A. Hannan, Yasser H. A. Abdel-Wahab

**Affiliations:** 1 Department of Pharmacy, School of Pharmacy and Public Health, Independent University, Bangladesh (IUB), Dhaka 1229, Bangladesh; akib_pharmspph@iub.edu.bd (A.B.R.); abdul.aziz@iub.edu.bd (M.A.A.); richichowdhury98@gmail.com (A.E.R.); ayesharahman32@gmail.com (A.R.); ummejafrin112000@gmail.com (U.H.J.); jmahannan@iub.edu.bd (J.M.A.H.); 2 School of Biomedical Sciences, Ulster University, Coleraine BT52 1SA, UK; y.abdel-wahab@ulster.ac.uk; 3 Department of Public Health, School of Pharmacy and Public Health, Independent University, Bangladesh (IUB), Dhaka 1229, Bangladesh; sam.t.chy@gmail.com; 4 Natural Products Research Laboratory, Strathclyde Institute of Pharmacy and Biomedical Sciences, University of Strathclyde, Glasgow G4 0RE, UK; veronique.seidel@strath.ac.uk

**Keywords:** quercetin, diabetes, inflammatory markers, medicinal plants, insulin

## Abstract

Diabetes Mellitus (DM) is a metabolic disorder that is spreading alarmingly around the globe. Type-2 DM (T2DM) is characterized by low-grade inflammation and insulin resistance and is closely linked to obesity. T2DM is mainly controlled by lifestyle/dietary changes and oral antidiabetic drugs but requires insulin in severe cases. Many of the drugs that are currently used to treat DM are costly and present adverse side effects. Several cellular, animal, and clinical studies have provided compelling evidence that flavonoids have therapeutic potential in the management of diabetes and its complications. Quercetin is a flavonoid, present in various natural sources, which has demonstrated in vitro and in vivo antidiabetic properties. It improves oral glucose tolerance, as well as pancreatic β-cell function to secrete insulin. It inhibits the α-glucosidase and DPP-IV enzymes, which prolong the half-life of glucagon-like peptide-1 (GLP-1) and glucose-dependent insulinotropic polypeptide (GIP). Quercetin also suppresses the release of pro-inflammatory markers such as IL-1β, IL-4, IL-6, and TNF-α. Further studies are warranted to elucidate the mode(s) of action of quercetin at the molecular level. This review demonstrates the therapeutic potential of quercetin in the management of T2DM.

## 1. Introduction

Diabetes mellitus (DM) is a chronic disease that is one of the leading causes of illness and mortality across the globe. DM is diagnosed as a result of an elevated blood glucose level (hyperglycaemia) caused by inadequate insulin secretion, defective insulin action, or both. The improper control of insulin has also been linked to abnormalities in the metabolism of lipids and proteins. If proper treatment is not received on time, or if left untreated, DM can lead to hyperglycaemic coma, and severe damage to the eyes, kidneys, blood vessels, and nervous and cardiovascular system. It can even lead to death due to ketoacidosis and nonketotic hyperosmolar syndrome [1,2]. These metabolic disruptions result from low insulin levels or insulin resistance in skeletal muscles, adipose tissue, and other target tissues. The development, pathogenesis, and complications of DM have been strongly correlated with high levels of oxidative stress, free radicals, and other metabolic stressors [3,4]. According to reports from 2021, 465 million people suffer from DM worldwide [5]. This number is anticipated to rise to 700 million by 2045. The majority of DM sufferers are from middle and low-income countries [5].

The American Diabetes Association has categorized diabetes as Type 1, Type 2, and gestational DM [6]. Type 1 diabetes, also known as juvenile diabetes, causes a decrease in glucose sensitivity to clonal pancreatic β-cells [7]. It has no cure but can be controlled by lifestyle changes, blood sugar monitoring, and the administration of insulin. This type of diabetes occurs in approximately 80–90% of children and adolescents [8]. Type 2 diabetes mellitus (T2DM) is the most prevalent and occurs due to the insufficient production of insulin by the body, insulin resistance, and obesity [9]. It can be controlled by lifestyle/dietary changes and oral antidiabetic drugs but requires insulin in severe cases [10]. Whilst the majority of T2DM sufferers are adults (more than 90% of the patient population), it affects people of all ages. Individuals over 40 years of age and with obesity issues and a family history of the disease are at a higher risk of developing T2DM [11].

A range of antidiabetic drugs such as metformin, sulfonylureas, meglitinides, thiazolidinediones, GLP-1 mimetics, DPP-IV, and SGLT2 inhibitors are currently used to treat T2DM. However, many of these are costly and present notable adverse side effects (Table 1) [12]. Plant-based medicines have emerged as an alternative treatment for DM, particularly as these are affordable, widely accessible to rural populations, and have been associated with low side effects [13,14]. According to the World Health Organization, 75% of the world’s population uses herbal medicine for basic healthcare needs [14]. Traditional medicinal plants are often used for a wide variety of ailments, including DM [15]. Phytoconstituents from medicinal plants are well-known for their valuable therapeutic potential owing to their various biological effects including antidiabetic, anti-inflammatory, cardioprotective, antiviral, and antibacterial activities [16]. Flavonoids, including quercetin, are found in several medicinal plants including *Momordica charantia*, *Dracocephalum moldavica*, *Euphorbia helioscopia*, and *Brassica rapa* (Table 2). Medicinal plants containing quercetin have been used traditionally for the treatment of diabetes, infections, and cancer [16]. Recent studies have revealed that quercetin reduces the risk of cardiovascular diseases by lowering hyperglycaemia, high blood pressure, hyperlipidaemia, and promoting weight loss [17]. Some studies have demonstrated that this flavonoid is beneficial in chronic hypertension, dyslipidemia, obesity, and T2DM [18]. Quercetin has been proven to decrease blood glucose, liver glucose content, and enzyme levels, and lower serum cholesterol levels [18,19]. It has also been shown to prevent oxidative damage, enhancing the regeneration of pancreatic β-cell islets, and the subsequent release of insulin [19]. In comparison to current synthetic drugs, which have many adverse effects, quercetin has proven to be an exceptional template for the development of novel antidiabetic drugs. The purpose of this review is to explore the therapeutic potential of quercetin in the management of T2DM.

## 2. Chemistry of Quercetin

**Figure 1:**
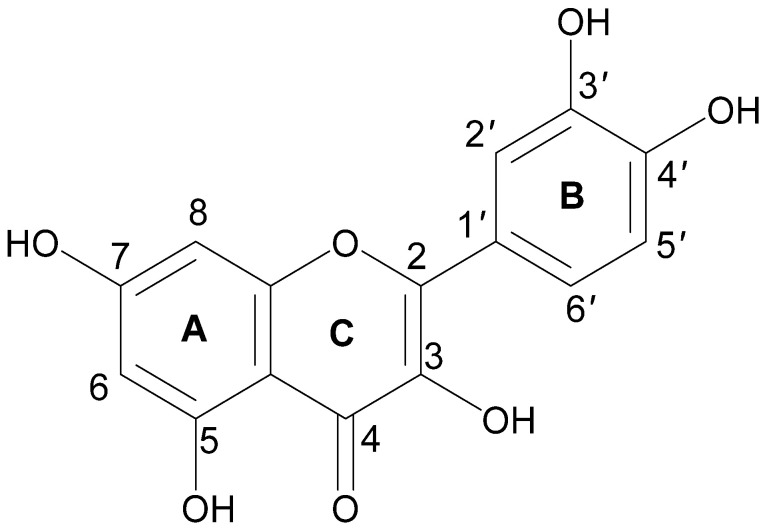
Chemical structure of quercetin with (A, B) and (C) representing the aromatic and γ-pyrone rings, respectively.

The term quercetin is derived from the Latin word “Quercetum” which means oak forest. The main dietary sources of quercetin are fruits, vegetables, and various medicinal plants (Table 2). Quercetin (3,3′,4′,5,7-pentahydroxyflavone) is a compound yellow in color, fully soluble in lipids and alcohol, insoluble in cold water, and sparingly soluble in hot water, that was isolated as a flavonoid glycoside for the first time in 1854. Its chemical structure was elucidated in 1899 [37]. Quercetin belongs to the flavonol subclass of flavonoids, with two aromatic rings (A and B) interlinked by a three-carbon linked γ-pyrone ring (C), and five hydroxyl (OH) groups that can be variously substituted (Figure 1).

The majority of quercetin derivatives are found in a glycoside form in which one or more hydroxyl group is substituted by different types of sugars [38]. Its polyphenolic structure, catechol moiety in the B ring, OH groups at positions 3 and 5 in the A ring, and 2,3-double bond conjugated with a 4-oxo function in the C ring have been identified as important features responsible for the well-known antioxidant effect of quercetin [39,40].

## 3. Pharmacological Actions of Quercetin in Diabetes and Associated Metabolic Disorders

**Figure 2:**
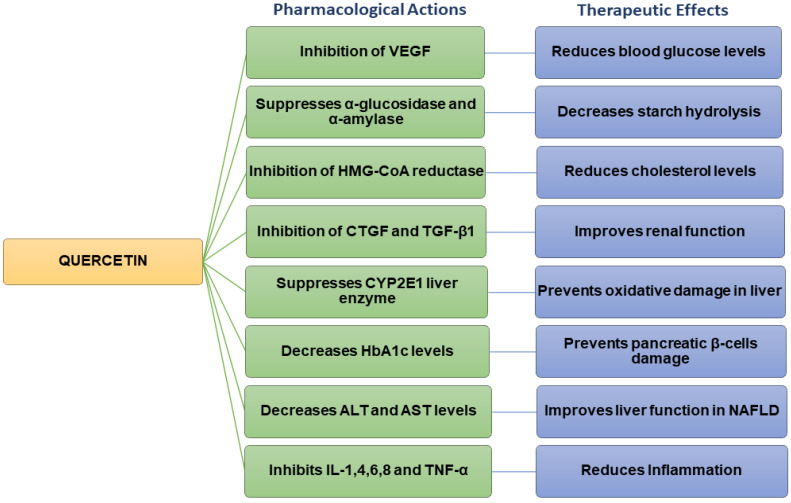
Flow chart summarizing the pharmacological actions and therapeutic effects of quercetin.

Quercetin possesses various pharmacological properties and has been reported as one of the most widely used flavonoids to treat metabolic and inflammatory disorders [14]. In vitro studies on human retinal endothelial cells demonstrated that quercetin could inhibit the proliferation of high-glucose-induced cells by lowering the production of vascular endothelial growth factor (VEGF) (Figure 2) [41]. Quercetin also inhibited carbohydrate digesting-enzymes (intestinal α-glucosidase and pancreatic α-amylase), reduced starch hydrolysis, decreased the rate of glucose absorption, as well as slowed down the progression of postprandial hyperglycaemia in in vitro settings (Figure 2) [42,43].

Studies carried out on streptozotocin (STZ)-induced diabetic rats have revealed that quercetin could reduce blood glucose levels and improve glucose tolerance [44]. Quercetin decreased plasma glucose levels in Type 2 diabetic rats [45]. In hyperlipidaemic animals, quercetin lowered the levels of triglycerides (TG), total cholesterol (TC), LDL, and VLDL cholesterol, inhibited 3-hydroxy-3-methylglutaryl-CoA (HMG-CoA) reductase, and increased adiponectin and HDL cholesterol levels (Table 2) [45,46]. Previous findings also indicated that quercetin could improve the high-fat diet (HFD)-induced dyslipidaemia in Swiss albino mice [47]. Other studies have shown that quercetin inhibited the overexpression of connective tissue growth factor (CTGF) and transforming growth factor beta-1 (TGF-β1) and contributed to improving renal function in diabetic nephropathic rats (Figure 2) [48].

Quercetin has the potential to prevent diabetic liver oxidative damage by suppressing the CYP2E1 liver enzyme in diabetic mice (Figure 2) [49]. Additionally, it decreases oxidative stress in diabetic renal tissue (Table 2) [50]. The administration of quercetin decreased body weight, fat accumulation, hyperglycaemia, dyslipidemia, and hyperinsulinemia in high-fat-fed obese mice [50]. Quercetin reduced blood glucose levels in mice and rats with T2DM [45,51]. It also decreased oxidative damage in the pancreatic tissue of high-fat-fed mice [52]. When combined with resveratrol, quercetin significantly upregulated gene-associated glucose or lipid metabolism, as well as liver function, in HFD animal models [53]. Furthermore, quercetin with/without resveratrol reduced the damage to pancreatic β-cells by restoring serum C-peptide and glycosylated hemoglobin (HbA1c) levels in diabetic rats (Figure 2) [54]. Histological investigations demonstrated that quercetin, with/without resveratrol, preserved pancreatic tissue and regulated insulin levels, thereby exerting hypoglycemic activity, and enhancing the function of pancreatic β-cells in diabetic rats [55]. Quercetin reduced serum lipids levels and showed beneficial effects on dyslipidemia-associated complications including atherosclerosis, myocardial attack, and coronary diseases [50]. Quercetin significantly decreased plasma glucose levels in STZ-induced diabetic rats. In addition, it improved glucose tolerance and hepatic glucokinase activity [44]. Quercetin increased the number of pancreatic islets in both normal and diabetic mice. It also regenerated the pancreatic islets and enhanced insulin secretion in STZ/alloxan-induced diabetic mice [56].

In a randomized, double-blind, placebo-controlled clinical trial, quercetin at a dose of 100 mg/day for 12 weeks reduced body fat and body mass index (BMI) of obese subjects [57]. It also downregulated triacylglycerol levels at a dose of 150 mg/day in overweight individuals [57]. Quercetin also lowered maltose-induced postprandial hyperglycaemia but had no significant effect on glucose-induced postprandial hyperglycaemia [58]. The oral administration of multiple doses of quercetin decreased blood glucose and HbA1c levels, enhanced glycogen synthesis, decreased α-glucosidase activity, and insulin resistance. In addition, it minimized β-cell insufficiency, enhancing pancreatic insulin secretion and controlling blood glucose levels in diabetic patients by reducing oxidative stress [59].

## 4. Other Activities and Side Effects of Quercetin

Due to its polyphenolic structure and its catechol moiety, quercetin displays antioxidant/radical scavenging properties [60,61]. These effects help to protect against the oxidative stress-induced damage to pancreatic β-cells associated with diabetes [62]. In addition, quercetin has cardioprotective, anti-tumour, anti-arthritis, and antimicrobial properties [63,64,65,66] and it also prevents tyrosinase enzyme activity [67]. Quercetin has been reported to treat non-alcoholic fatty liver disease (NAFLD) by decreasing the level of liver enzymes, such as alanine transaminase (ALT) and aspartate transaminase (AST) (Figure 2), oxidative stress, and inflammation, and by regenerating altered metabolites and gut microbiota [44,68]. Quercetin has shown immunomodulatory activity, reducing the release of pro-inflammatory cytokines such as interleukin (IL)-1, IL-6, IL-8, IL-4, and tumour necrosis factor (TNF)-α (Figure 2) [69]. At doses higher than 945 mg/m^2^, quercetin can cause emesis, hypertension, nephrotoxicity, and decrease serum potassium levels [46]. Quercetin was also found to increase insulin secretion from BRIN-BD11 cells in a dose-dependent manner; however, it showed toxicity at doses above 50 μM [15].

**Table 2:** Pharmacological actions of quercetin-containing plants.

Plant Names	Plant Part(s)	Diabetic Model/s	Pharmacological Actions of Plants	Dose of Quercetin	Duration of Treatment	Pharmacological Actions of Quercetin	References
*Acanthopanax senticosus*	Root	Alloxan-induced diabetic rats	↓ Blood glucose, total cholesterol, total bilirubin, creatinine, urea ↓ Oxidative stress	50 mg/kg	30 days	Inhibits α-glucosidase activity Reduces oxidative stress	[66,70,71]
*Ginkgo biloba*	Leaf	STZ-induced diabetic rats	↑ β-cell mass and insulin secretion ↓ Amyloid-β neurotoxicity	90 mg/kg	10 weeks	Delays the progression of STZ-induced diabetic cataracts Reduces AGE products activity	[72,73]
*Psidium guajava*	Leaf	NA-STZ-induced diabetic rats	↓ Oxidative stress ↓ Protein glycation ↓ Inflammation	10- 50 mg/kg	28 days	Reduces blood glucose levels Increases insulin secretion Improves T2DM-mediated cardiovascular disease	[74,75]
*Momordica charantia*	Fruit	HFF obese rats	↓ Blood glucose, total cholesterol ↑ Insulin secretion	50 mg/kg	12 weeks	Reduces oxidation stress by inhibiting the release of chemokines and cytokines	[76,77]
*Polygonum perfoliatum*	Leaf	HFF obese rats	↓ Blood glucose ↓ Inflammation	60–240 mg/kg	4 weeks	Inhibits α-glucosidase activity	[78]
*Phyllanthus Emblica*	Fruit	STZ-induced diabetic rats	↓ Triglycerides, LDL, VLDL, total cholesterol ↑ HDL cholesterol	25–75 mg/kg	28 days	Decreases blood glucose Increases insulin secretion	[79]
*Cuscuta chinensis*	Seed	Alloxan-induced diabetic mice	↓ Fasting blood glucose ↑ Insulin secretion Inhibits DPP-IV activity	20 mg/kg	3 weeks	Reduces fasting blood glucose level Enhances GLUT4 expression	[65,80]
*Euphorbia helioscopia*	Leaf, root	STZ-induced diabetic rats	↑ Insulin secretion ↓ Blood glucose	100 mg/kg	7 weeks	Reduces blood glucose and blood glycated hemoglobin levels	[81,82]
*Brassica rapa*	Root	STZ-induced diabetic rats	↓ Fasting blood glucose ↓ Inflammation ↓ Hypertension Inhibits DPP-IV activity	15 mg/kg	25 days	Decreases blood glucose levels Improves glucose tolerance	[83,84]
*Crataegus pinnatifida*	Leaf, fruit	STZ-induced diabetic rats	↓ Fasting blood glucose ↓ VLDL and LDL cholesterol	100 mg/kg	14 days	Decreases blood glucose Increases plasma insulin	[85,86]
*Sophora japonica*	Bud, flower	STZ-induced diabetic rats	↑ Insulin release Inhibits DPP-IV activity	10–15 mg/kg	10 days	Reduces blood glucose levels Improves glucose tolerance	[50,87]
*Coriandrum sativum*	Herb	STZ-induced diabetic rats	↑ Insulin secretion ↓ Blood glucose ↓ Inflammation	50 mg/kg	8 weeks	Decreases fasting blood glucose Suppresses TNF-α, IL-1β, and production of AGEs	[88,89,90]
*Cymbopogon citratus*	Herb	STZ-induced diabetic rats	↓ Fasting blood glucose ↓ Inflammation ↓ Hypertension ↑ Insulin secretion	20–50 mg/kg	6 weeks	Reduces blood glucose levels Decreases the production of reactive oxygen species (ROS) Improves T2DM-mediated testicular damage	[91,92,93]
*Allium cepa*	Bulb	STZ-induced diabetic rats	↓ Blood glucose ↓ Triglycerides, LDL, VLDL, total cholesterol ↑ HDL cholesterol ↑ Insulin secretion	100–200 mg/kg	6 weeks	Lowers blood glucose Improves glucose tolerance	[94,95,96]
*Prunus avium*	Fruit	STZ-induced diabetic rats	↓ Blood glucose ↑ Insulin secretion ↓ LDL and VLDL cholesterol	50–80 mg/kg	45 days	Reduces blood glucose levels Improves oxidative stress	[97,98,99]
*Capparis spinosa*	Fruit	Alloxan-induced diabetic mice	↓ Fasting blood glucose ↑ Insulin secretion ↓ Liver damage	50 mg/kg	7 days	Decreases fasting blood glucose Reduces ALT and AST levels	[100,101,102]
*Brassica oleracea var. Italica*	Flower	STZ-induced diabetic rats	↑ Insulin secretion ↓ Blood glucose	10 mg/kg	4 weeks	Decreases blood glucose levels Reduces creatinine and blood urea nitrogen levels	[103,104,105]
*Lactuca sativa*	Leaf	Alloxan-induced diabetic rats	↓ Fasting blood glucose ↑ Insulin secretion ↓ Inflammation	50 mg/kg	4 weeks	Reduces blood glucose levels Decreases creatinine, ALT, AST, and cholesterol levels	[106,107,108]
*Asparagus officinalis*	Stem	STZ-induced diabetic rats	↑ Insulin secretion ↓ Blood glucose ↓ Inflammation	50 mg/kg	12 weeks	Reduces fasting blood glucose Decreases the production of reactive oxygen species (ROS) Improves glucose tolerance	[109,110]
*Acacia arabica*	Bark	HFF-induced obese diabetic rats	↑ Insulin secretion Inhibits DPP-IV activity ↓ Protein glycation	30 mg/kg	8 weeks	Reduces fasting blood glucose Decreases LDL and TG levels Increases HDL levels	[15,111]
*Solanum lycopersicum*	Fruit	STZ-induced diabetic rats	↓ Blood glucose ↑ Insulin secretion	10 mg/kg	28 days	Decreases blood glucose levels Increases insulin secretion Inhibits apoptosis	[112,113]
*Piper nigrum*	Flower	Alloxan-induced diabetic mice	↑ Insulin secretion ↓ Blood glucose ↓ Inflammation	50 mg/kg	7 days	Reduces blood glucose levels	[114,115]
*Toona sinensis*		HFF-induced obese diabetic rats	↑ Insulin secretion ↓ Blood glucose ↓ Inflammation	200 mg/kg	4 weeks	Improves glucose tolerance Decreases TG and TC levels	[116]

Symbols. ↑: Increase; ↓: Decrease.

## 5. Mechanisms of Action of Quercetin

**Figure 3:**
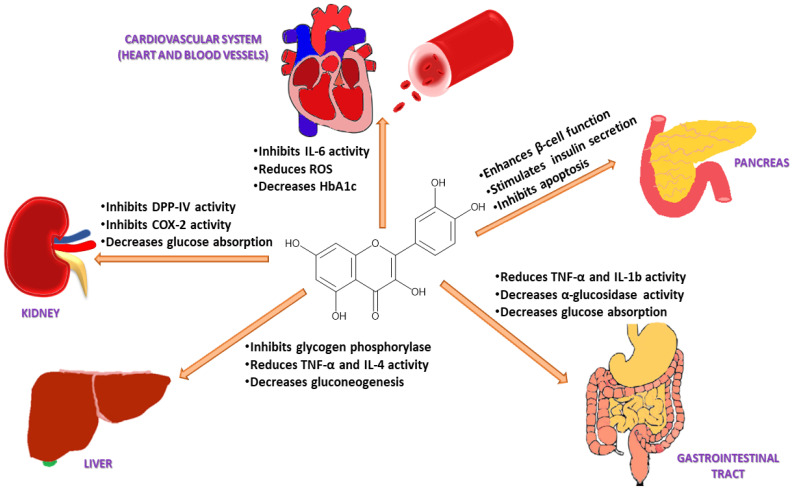
Pharmacological action of quercetin via different mechanistic pathways: Quercetin enhances pancreatic β-cell function and increases insulin release by inhibiting apoptosis, NF-κB, and JNK pathways; decreases glucose absorption in the kidney by inhibiting DPP-IV and COX-2 activity; decreases gluconeogenesis through inhibition of TNF-α and IL-4 in the liver; suppresses glucose reabsorption in the gastrointestinal tract by decreasing α-glucosidase activity; reduces blood glucose levels and oxidative stress by inhibiting IL-6 activity in the heart and blood vessels.

Quercetin maintains glucose homeostasis by interacting with molecular targets in the small intestine, pancreas, skeletal muscle, adipose tissue, and liver. Studies carried out on STZ-induced diabetic rats have revealed that quercetin could restore the impaired protein expression of insulin signaling molecules, such as phosphatidylinositol 3 kinases (PI3K) and insulin receptor substrate-1 (IRS-1), resulting in increased insulin-mediated glucose uptake [117]. Quercetin has also been shown to activate adenosine monophosphate-activated protein kinase (AMPK) in the livers of rats, which reduces glucose synthesis primarily via downregulating glycogenic isoenzymes, such as phosphoenolpyruvate carboxylase (PEPCK) and glucose-6-phosphatase (G6Pase) [52,118]. In mouse skeletal muscle cells, it has been reported to enhance glucose uptake by promoting the translocation of GLUT4 to the cell membrane [119]. These findings indicate that quercetin regulates the metabolism of glucose, increasing glycolysis while decreasing gluconeogenesis [120]. In healthy individuals, around 80% of the absorbed glucose is stored in the form of glycogen in skeletal muscles upon the action of insulin. A reduction in this uptake has been shown to contribute to the etiology of T2DM as irregularities in the expression of the GLUT4 transporter lower the rate of glucose entering the cells, leading to a rise in blood glucose levels [121]. In skeletal muscles, quercetin activates AMPK, which in turn stimulates GLUT4 receptors and Akt (protein kinase B) in the cell membrane [122]. This allows glucose to enter the cells via the GLUT4 transporter, thereby regulating glycaemia [117]. Similarly, exercise is a potent activator of GLUT4 expression, which increases insulin activity and muscle glycogen storage. Defective activation of AMPK leads to insulin resistance, which causes T2DM [123]. Quercetin-induced AMPK activation in hepatocytes inhibits glucose-6 phosphatases [118]. Treatment with quercetin decreases GLUT2 expression and the intestinal sodium-dependent glucose uptake, in turn reducing glucose absorption in the gastrointestinal tract and controlling glycaemia (Figure 3) [119].

In addition, quercetin has been reported to enhance the AMP/ATP ratio and scavenge reactive oxygen species (ROS) in clonal pancreatic β-cells, reducing oxidative stress (Figure 3) [124]. The increased AMP/ATP ratio activates the mitochondrial target of rapamycin (mTOR), which induces mitogenesis (via transduction signaling pathways activation) and stimulates insulin secretion [125]. mTOR plays a significant role in the regulation of transcription, protein synthesis, and cell nutrition [126]. Under hyperglycaemic conditions, proteins, lipids, and nucleic acids undergo non-enzymatic glycation to form Advanced Glycation End (AGE)-products. The latter cause diabetic complications such as cardiovascular diseases, nephropathy, retinopathy, and neuropathy. Quercetin has been found to inhibit protein glycation more potently than the synthetic drug aminoguanidine [122,123]. It can reduce the formation of AGE-products by trapping methylglyoxal and glyoxal [127] and improve diabetic complications due to its antioxidant, anti-inflammatory, and antihyperglycemic properties [47].

In STZ-induced rats, quercetin improved retinopathy by down-regulating matrix metalloproteinase-9 (MMP-9), monocyte chemo-attractant protein-1 (MCP-1), and vascular endothelial growth factor (VEGF) [128]. In hypercholesterolemic mice, it reduced diabetic nephropathy by lowering triglycerides and blood glucose levels [129]. Moreover, it also showed neuroprotective effects on enteric neurons in the cecum of DM rats [130].

Previous studies have revealed that fat accumulation in the liver and muscles activates the Jun N-terminal kinases (JNK) and the nuclear transcription factor Kappa-B (NF-κB) inflammatory pathways, leading to obesity-associated T2DM (Figure 3) [131]. Quercetin suppresses both these pathways, which in turn improves glycaemia [132]. It also suppresses the FcεRI receptor by inhibiting the phosphorylation of several kinases like PKC (protein kinase C), Syk (spleen tyrosine kinase), and p38 mitogen-activated protein kinase (MAPK) in mast cells and basophils [122].

The release of pro-inflammatory mediators such as IL-1, IL-6, IL-8, IL-4, TNF-α, and histamine in brown adipose tissue has been linked with increased insulin resistance and high blood glucose levels (Figure 3) [133]. Quercetin inhibits these mediators and prevents oxidative stress [134]. In the kidneys, quercetin reduces DPP-IV and cyclooxygenase-2 (COX-2) activity, leading to a decrease in blood glucose reabsorption (Figure 3) [135]. Quercetin also activates leukocytes and targets various enzymes such as kinases, membrane proteins, and phosphatases to control inflammation and the immune response [129]. It suppresses lipoxygenase and cyclooxygenase enzymes, which suppresses the release of pro-inflammatory mediators including leukotrienes and prostaglandins [136]. Quercetin also inhibits TNF-α, a cytokine that plays a vital role in leukocyte formation, proliferation, and differentiation, specifically in the liver and gastrointestinal tract [69,125,135]. This causes a reduction in gluconeogenesis, glucose reabsorption, and α-glucosidase activity (Figure 3) [135]. Pancreatic β-cell apoptosis may occur due to hyperglycaemia-induced oxidative stress, and this can lead to diabetes mellitus. Glutathione peroxidase 4 (GPX4), an enzyme that protects cells against lipid peroxidation, suppresses the ferroptosis or apoptosis of pancreatic β-cells [137]. It has been demonstrated that quercetin can increase GPX4 activity in the pancreas, reducing oxidative stress, increased β-cell production, and insulin secretion [138]. Quercetin has also been reported to reduce the intestinal absorption of cholesterol by reducing the expression of the epithelial cholesterol transporter Niemann-Pick C1-Like 1 (NPC1L1) [100] and it has been suggested that the consumption of quercetin in the diet could lower systolic, diastolic, and mean arterial pressure in hypertensive individuals [139]. Quercetin can also lower blood pressure by reducing oxidative stress, enhancing the renin-angiotensin-aldosterone system (RAAS), and increasing vascular activity [133].

## 6. Effects of Quercetin on Diabetic Complications

Hyperglycemia over an extended period of time can increase the risk of macro- and microvascular problems including retinopathy, nephropathy, neuropathy, and cardiovascular diseases. A serious complication of DM, diabetic retinopathy (DR) is one of the leading causes of adult blindness and visual impairment [140]. Recent studies have indicated that quercetin at 150 mg/kg improved retinopathy in STZ-induced rats by reducing the expression of monocyte chemoattractant protein-1 (MCP-1), matrix metalloproteinase-9 (MMP-9), and vascular endothelial growth factor (VEGF), and decreased protein damage caused by oxidative stress [49,141]. Another major complication of DM, diabetic nephropathy is caused by long-term hyperglycemia which leads renal cells to secrete an array of pro-inflammatory and pro-fibrotic substances, resulting in cell hypertrophy and proliferation and the development of renal interstitial fibrosis [142]. Previous reports have shown that quercetin deactivated the SphK1-S1P (sphingosine kinase-1) signaling pathway, and hence inhibited the development of renal fibrosis [48].

Oxidative stress due to chronic hyperglycaemia induces complications related to the central nervous system and thus, may lead to neurodegenerative disorders such as Parkinson’s and Alzheimer’s diseases [143]. Recent studies have shown that the administration of quercetin improved memory impairment and reduced brain energy metabolism in STZ-induced rats by reducing ATP content in a dose-dependent manner [144]. Chronic insulin resistance and hyperglycaemia increase the risk of macrovascular complications such as hypertension, cardiomyopathy, and coronary artery diseases [145,146]. In STZ-induced rats, quercetin, with or without glibenclamide, was seen to decrease damage due to cardiomyopathy, in a dose-dependent manner [146]. Recent studies have revealed that quercetin increased cardioprotection via increasing endothelium cell receptors and nitric oxide production in STZ-induced rats [147]. Another study performed with Type 2 diabetic women reported that quercetin supplements could significantly reduce systolic blood pressure [148].

In recent years, various studies have reported the positive effects of quercetin on diabetes and its complications, and it is likely that these are alleviated following quercetin administration. However, few of these studies have been performed on humans. In recent years, only a few antidiabetic mechanisms of action have been reported for quercetin. More clinical trials including those using quercetin supplementation are required to better understand the mechanism(s) of action of quercetin in humans. It has also been discovered that defective iron metabolism in diabetic patients leads to increased severity of diabetic complications [149]. However, the impact of quercetin on iron regulation in diabetic complications has rarely been reported. Further investigation is required to understand the role of quercetin in iron regulation to develop new potential drugs for nephropathy, neuropathy, retinopathy, and other diabetic complications.

## 7. Conclusions

Studies have revealed that quercetin displays a wide range of pharmacological properties, including antihyperglycaemic effects. It can alleviate hyperglycaemia, hyperlipidemia, hypertension, and oxidative stress, contributing to lowering the risk of cardiovascular diseases emerging. Quercetin decreases blood glucose levels, improves glucose tolerance, and enhances pancreatic β-cell function via various mechanistic pathways such as AMPK which regulates GLUT4 expression in adipose tissue and muscles. It also regulates glycaemia by reducing GLUT2 expression and sodium-dependent glucose uptake in the gut, as well as lowering glucose absorption. It also inhibits the release of pro-inflammatory mediators, such as TNF-α, IL-1, -4, -6, and -8, preventing pancreatic β-cell damage. Quercetin has been shown to improve insulin sensitivity, glucose metabolism, and insulin secretion in diabetic animal models by promoting pancreatic β-cell proliferation. Due to its numerous benefits, quercetin has been identified to play a vital role in the treatment of T2DM. Various studies are currently underway to determine the potential of quercetin as a future antidiabetic medicine. However, only a few clinical trials have been performed to understand how quercetin works in humans. Therefore, the correct dose and duration of quercetin treatment are still unknown. It is necessary to address these limitations in current and future studies in order to confirm the true effects of quercetin on diabetic patients. The studies presented in this review support the conclusion that quercetin is a promising template for the development of new antidiabetic drugs. These would offer an alternative to current synthetic drugs that have undesirable side effects. However, further studies, ranging from animal models to clinical trials, are warranted to investigate the effects, including the mechanism(s) of action at the molecular level, of quercetin on lowering blood glucose levels and increasing insulin release in T2DM.

## Figures and Tables

**Table 1:** Pharmacological actions and side effects of antidiabetic drugs.

Type 2 Antidiabetic Agents	Pharmacological Actions	Side Effects	References
α-glucosidase inhibitors (Acarbose, miglitol)	Inhibit the intestinal absorption of carbohydrates	Flatulence, bloating, diarrhoea	[20,21]
Biguanides (Metformin)	Inhibit hepatic gluconeogenesis, Reduce the liver and intestinal absorption of sugar Increase insulin sensitivity and glucose uptake	Kidney complications, upset stomach, tiredness, and dizziness	[22,23]
Dopamine agonists (Bromocriptine, cabergoline, apomorphine)	Regulate plasma glucose, free fatty acids, and triglyceride levels in insulin-resistant patients	Visual hallucinations and confusion, edema	[24,25]
Dipeptidyl peptidase-4 (DPP-4) inhibitors (Sitagliptin, saxagliptin, linagliptin)	Increase the half-life of GLP-1 and GIP	Gastrointestinal problems, flu-like symptoms (headache, runny nose, sore throat)	[26,27]
GLP-1 agonists (Dulaglutide, exenatide, albiglutide)	Enhance insulin release Reduce glucagon release	Gastrointestinal problems and nausea	[28,29]
Meglitinides (Nateglinide, repaglinide)	Stimulate the release of insulin	Weight gain, hypoglycaemia, excessive sweating	[30,31]
Sodium-glucose Co-transporter-2 (SGLT-2) inhibitors (Dapagliflozin, canagliflozin, empagliflozin)	Inhibit glucose reabsorption in the renal tubule	Urinary tract infection and increased urination, upper respiratory tract infections, joint pain, nausea, and thirst	[32,33]
Sulfonylureas (Tolbutamide, tolazamide, chlorpropamide)	Inhibit ATP-sensitive potassium (K_ATP_) channel in pancreatic β-cells	Hypoglycaemia, upset stomach, skin rash, and itching	[34]
Thiazolidinediones (Rosiglitazone, pioglitazone)	Bind with the peroxisome proliferator-activated receptor (PPAR)-γ receptor resulting in the activation of several genes that regulate glucose metabolism in the liver	Anaemia risk, weight gain, edema, heart failure	[35,36]

## Data Availability

Not applicable.

## Abbreviations

DM	Diabetes Mellitus
T2DM	Type 2 Diabetes Mellitus
DPP-IV	Dipeptidyl peptidase 4
GLP-1	Glucagon-like peptide-1
GIP	Glucose-dependent insulinotropic polypeptide
IL	Interleukin
TNF	Tumor necrosis factor
SGLT2	Sodium-glucose cotransporter-2
PPAR	Peroxisome proliferator-activated receptors
VEGF	Vascular endothelial growth factor
TG	Triglycerides
HDL	High-density lipoprotein
LDL	Low-density lipoprotein
VLDL	Very low-density lipoprotein
HMG-CoA	3-hydroxy-3-methylglutaryl coenzyme A
HFD	High fat fed
CTGF	Connective tissue growth factor
TGF-β1	Transforming growth factor-β1
CYP2E1	Cytochrome P450 2E1
BMI	Body mass index
NAFLD	Non-alcoholic fatty liver disease
NA	Nicotinamide
ALT	Alanine transaminase
AST	Aspartate transaminase
STZ	Streptozotocin
AGE	Advanced Glycation End Products
IRS-1	Insulin receptor substrate 1
AMPK	AMP-activated protein kinase
PEPCK	Phosphoenolpyruvate carboxykinase
G6Pase	Glucose 6 Phosphate
Akt	Protein kinase B
AMP	Adenosine monophosphate
ATP	Adenosine triphosphate
ROS	Reactive oxygen species
MMP-9	Matrix metalloproteinase 9
MCP-1	Monocyte chemoattractant protein-1
JNK	c-Jun N-terminal kinase
NF-κB	Nuclear transcription factor kappa-B
PKC	Protein kinase C
Syk	Spleen tyrosine kinase
MAPK	p38 mitogen-activated protein kinase
COX-2	Cyclooxygenase-2
GPX4	Glutathione peroxidase 4
NPC1L1	Niemann-Pick C1-Like 1
RAAS	Renin-angiotensin-aldosterone system
